# Lung Volume Change Under Apnoeic Oxygenation With Different Flow Rates in Children: A Single‐Centre Prospective Randomized Controlled Non‐Inferiority Trial

**DOI:** 10.1111/pan.70018

**Published:** 2025-07-18

**Authors:** Jonas Aebli, Vera Bohnenblust, Gabriela Koepp‐Medina, Sara Ahsani‐Nasab, Markus Huber, Robert Greif, Nicola Disma, Thomas Riva, Thomas Riedel, Alexander Fuchs

**Affiliations:** ^1^ Department of Anesthesiology and Pain Medicine Inselspital, Bern University Hospital, University of Bern Bern Switzerland; ^2^ Unit of Biostatistics, Epidemiology and Public Health, Department of Cardiac, Thoracic, Vascular and Public Health University of Padova Padova Italy; ^3^ Medical Faculty University of Bern Bern Switzerland; ^4^ Department of Surgical Science University of Torino Torino Italy; ^5^ Unit for Research in Anesthesia IRCCS Istituto Giannina Gaslini Genoa Italy; ^6^ Division of Pediatric Intensive Care Medicine, Department of Pediatrics Bern University Hospital, Inselspital, University of Bern Bern Switzerland

**Keywords:** airway pressure, anesthesia, apnoeic oxygenation, high‐flow oxygen, lung volume, pediatrics, respiratory physiology

## Abstract

**Background:**

High‐flow oxygen in children prolongs the apnea time. The exact mechanism remains unclear.

**Aims:**

This study investigated whether low‐ and high‐flow nasal oxygen are non‐inferior to very high‐flow oxygen in preventing lung volume loss during apnoea in children under general anesthesia. We also examined whether early onset oxygen using the Optiflow Switch cannula reduces lung volume loss compared to conventional late‐onset application. Finally, we assessed the timing and regional distribution of lung volume changes using electrical impedance tomography (EIT).

**Methods:**

We conducted a single center randomized controlled non‐inferiority trial. After Ethics Committee approval and informed consent, we recruited 108 children (ASA1 and 2, 10–20 kg) undergoing elective general anesthesia. The primary endpoint was the normalized reduction in lung volume in relation to body weight (mL kg^−1^) after termination of facemask ventilation from start to end of apnoea measured with EIT. After induction of anesthesia and neuromuscular blockade, patients were left apnoeic for 5 min receiving humidified and heated oxygen with a high‐flow system at different flow rates: (1) Low‐flow 0.2 L min^−1^ kg^−1^; (2) High‐flow 2 L min^−1^ kg^−1^; (3) Very high‐flow 4 L min^−1^ kg^−1^(control group); (4) Early onset of high‐flow 2 L min^−1^ kg^−1^ with Optiflow Switch. Normalization of impedance change to 6–8 mL kg^−1^ in relation to body weight and changes in lung volume from start to end of apnoea were measured.

**Results:**

89/108 children were analyzed (low‐flow *n* = 20, high‐flow *n* = 24, very high‐flow *n* = 21 and early onset high‐flow *n* = 24.). The estimated mean (95% CI) reduction in lung volume was: low‐flow 5.9 (5.3–7.8) mL kg^−1^, high‐flow 6.5 (5.3–7.8) mL kg^−1^, very high‐flow (control) 5.7 (4.4–7.0) mL kg^−1^, and early onset high‐flow 6.7 (5.5–7.9) mL kg^−1^. Non‐inferiority could be demonstrated only for the low‐flow group compared to the control group.

**Conclusions:**

Apnoeic oxygenation with low‐flow is non‐inferior to very high‐flow regarding lung volume loss in children. An early onset of apnoeic oxygenation after facemask ventilation may delay lung volume loss during apnoea.

## 1 Introduction

High‐flow nasal oxygen (HFNO) is the administration of blended air/oxygen heated and humidified via nasal cannula [1]. Due to its ease of use and safety, HFNO is gaining popularity and proving effective in various indications and settings. In pediatric intensive care units, it is used to treat bronchiolitis [2], respiratory distress [3] and as respiratory support following extubation [4].

Children desaturate rapidly during apnoea due to their high oxygen consumption and reduced functional residual capacity (FRC), making airway management more challenging [5, 6, 7, 8, 9, 10]. Supplementary oxygen during intubation delays the time to desaturation and prolongs the “safe” apnoea time [11, 12, 13], increases first attempt intubation success rates, and enables higher oxygen saturation throughout the procedure [14], likely making adverse events less common. Growing evidence suggests possible advantages of HFNO over low‐flow techniques for apnoeic oxygenation during intubation [15].

A positive subglottic pressure when using HFNO has also been suggested, considering that in spontaneously breathing adult and pediatric patients HFNO generates some increase in airway pressure [16, 17, 18, 19]. In contrast, a study on adult patients in apnoea showed that there was no relevant increase in airway pressure [20] or delay in atelectasis formation while patients mouths were open [21].

Currently, there is no data on pressure generated in the airway in apnoeic children treated with HFNO. The traditional measurement of intratracheal pressure with a catheter poses the risk of bronchospasm. A risk‐free alternative is thoracic electrical impedance tomography (EIT). Using a dynamic, cross‐sectional impedance measurement of the lung, changes in volume can be calculated and consequently give an indirect approximation of airway pressures [22].

The newly introduced Optiflow Switch nasal cannula (Fisher&Paykel, Auckland, New Zealand) consists of a flexible arm for oxygen inflow made of compressible soft material that can be easily pinched off. This nasal cannula can be used under a tightly fitted facemask, which permits the early onset of apnoeic oxygenation without delay after facemask removal. The use of this interface raises the question of whether it is possible to maintain greater positive airway pressure in the subglottic structures due to an immediate inflow of oxygen. This might potentially prevent the collapse of alveoli and therefore atelectasis formation. This trial was designed to explore three key aspects of apnoeic oxygenation in children undergoing general anesthesia. First, we sought to evaluate whether delivering oxygen at lower flow rates (0.2 and 2 L kg^−1^ min^−1^) is non‐inferior to a very high flow rate (4 L kg^−1^ min^−1^) in preventing lung volume loss. Second, we investigated whether an early onset of apnoeic oxygenation after facemask ventilation using the Optiflow Switch cannula provides any advantage in delaying or reducing lung volume loss. Third, we aimed to describe how lung volume changes evolve over time during apnoea and whether these changes differ regionally (anterior vs. posterior lung fields). These data could improve our understanding of applied respiratory physiology regarding the mechanisms underlying atelectasis formation in apneic pediatric patients. We hypothesized that there is no difference in lung volume loss regarding the flow rate and interface.

## 2 Methods

### 2.1 Ethics

After approval from the Cantonal Ethics Committee of Bern (KEK‐BE 2022–01833) and registration at ClinicalTrials.gov (NCT05672329), we obtained written informed consent from the parents or legal guardians of all children before study enrollment. This single‐centre, prospective, randomized controlled trial was conducted at the Department of Anaesthesiology and Pain Medicine at the Bern University Hospital, Bern, Switzerland, from January 2023 to April 2024.

### 2.2 Participants

We included pediatric patients requiring general anesthesia for elective surgery in the operating room. Inclusion criteria were body weight of 10–20 kg, American Society of Anesthesiology (ASA) physical status I and II, and written informed consent obtained from the legal guardians. The exclusion criteria were known or suspected difficult intubation, oxygen dependency, congenital heart or lung disease, BMI (kg m^−2^) > 30, and high aspiration risk.

Patients were randomly assigned to one of four equally sized intervention groups using a randomization software (www.sealedenvelope.com) by a study nurse not involved in the study. Concealment of randomization was guaranteed using sealed, opaque envelopes after adequate facemask ventilation was established. Blinding of the study team was not feasible due to the manner of oxygen administration and cannulas. Patients and parents were blinded to group allocation. Children were randomized to receive one of four different flow rates of 100% oxygen, heated and humidified by the high‐flow system:

1. Late onset low‐flow: 0.2 L kg^−1^ min^−1^
2. Late onset high‐flow group: 2 L kg^−1^ min^−1^
3. Late onset very high‐flow group (control): 4 L kg^−1^ min^−1^
4. Early onset high‐flow group: 2 L kg^−1^ min^−1^

The early onset group using the Optiflow Switch nasal cannula was introduced after the start of the study and enrolment of 8 patients, following formal clearance from the manufacturer to use the device in the studied pediatric age group. Once clearance was obtained, we performed a new randomization process with the remaining patients with addition of the Switch group. The integration of the new device required no additional training or procedural change, as the application of the Switch cannula followed the same principles as conventional HFNO and was familiar to the anesthesia team. Thus, no learning curve or operator‐related variability was expected.

### 2.3 Study Procedure

Children were premedicated with 0.5 mg/kg^−1^ midazolam (oral or rectal) or 2 μg kg^−1^ dexmedetomidine (nasal). Before the induction of anesthesia, all patients were monitored according to the local standard of anesthesia care. Transcutaneous CO₂ and EIT monitoring were initiated before induction (TCM 5, Radiometer, Krefeld. Germany; Pulmo Vista 500; Draeger, Luebeck, Germany). This belt consists of 16 electrodes plus one reference electrode and is fitted around the chest in a thoracic median plane. Intravenous (i.v.) access was established and induction was performed with fentanyl 2 μg kg^−1^ and propofol 2–3 mg kg^−1^. In case i.v. access was difficult to obtain, patients were induced with inhaled sevoflurane followed by the placement of an i.v. cannula. After induction, general anesthesia was maintained using infused propofol 10 to 15 mg/kg^−1^ h^−1^. Anesthetic depth was assessed with Narcotrend (Narcotrend, Hannover, Germany), maintaining values below 60. After induction, all patients were paralyzed with 0.9 mg/kg^−1^ of rocuronium. Neuromuscular block was assessed by train‐of‐four (TOF) monitoring using TOF‐Watch (Organon Ltd., Dublin, Ireland).

One minute of pressure‐controlled mask ventilation (P_max_ 20 cm H_2_O, PEEP 5cmH_2_0), with pressures adjusted to reach a volume of 6–8 mL kg^−1^ with 100% oxygen was applied. Meanwhile, we installed near‐infrared spectroscopy (NIRS; Nonin Medical inc., Plymouth, MN, USA). Before the start of the apnoeic period, TOF was confirmed to be zero. Then, the ventilation was discontinued, and the children were left apnoeic for 5 min with 100% O_2_, with the flow rate and device according to the randomization in a supine position. The gas mixture for HFNO was delivered using the Optiflow system (Fisher&Paykel, Auckland, New Zealand), which is composed of a humidifier base, a heater wire, a temperature probe, an oxygen/air blender, and a RT950 circuit that includes a humidifier chamber, tubing system, and a pressure relief valve with flow diverter. The adult nasal cannula or the Optiflow Switch in size *small* was used, taking care that the children's nostrils were not occluded more than 50%. Continuous jaw thrust and the insertion of an oropharyngeal airway (Guedel airway; Intersurgical, Wokingham, UK) ensured airway patency. The conventional high‐flow nasal cannula was installed and started right *after* pressure‐controlled facemask ventilation was discontinued. The Optiflow Switch cannula was installed and started *before* the 1 min of pressure‐controlled facemask ventilation. The apnoeic period ended after 5 min. If any of the following criteria were met, the apnoeic period was terminated immediately before reaching 5 min: (1) drop of SpO_2_ under 95%; (2) increase of transcutaneous CO_2_ above 70 mmHg; or (3) decrease of NIRS of more than 30% from baseline.

After 5 min of apnoea, facemask ventilation was resumed and planned airway management (e.g., tracheal intubation or supraglottic airway) was performed followed by a standardized lung recruitment maneuver.

A chest ultrasound (Venue Go; GE Healthcare, Chicago, Illinois, USA) after airway management ruled out a pneumothorax. Before discharge from the postanesthesia care unit (PACU) children were examined for any postoperative side effects such as coughing, bronchospasm, stridor, laryngospasm, pain, or postoperative nausea and vomiting.

### 2.4 Measurements

We recorded thoracic EIT measurements continuously at a frame rate of 30 Hz during the study period starting when the child was still breathing spontaneously before induction of anesthesia and ending 1 min after the recruitment maneuvers. We excluded patient data in cases of malfunction of the measurement device. We reconstructed EIT images from the raw data based on the Graz consensus reconstruction algorithm for electrical impedance tomography (GREIT) using the torso mesh function [23, 24]. The measurement of reduction in total lung volume was started after removal of the facemask when the apnoea started and was estimated by calculating lung impedance change, normalized to the impedance amplitude during mechanical ventilation at 6–8 mL/kg^−1^ using adapted customized code (Matlab R2021a; The MathWorks Inc., Nattick, MA, USA) [25, 26]. Additionally, the time to 25%, 50%, and 75% of total impedance change from baseline was calculated to detect potential differences in curve progression of the change in lung impedance. We performed all analyses for the global impedance signal and two regions of interest (anterior and posterior).

### 2.5 Statistics

The primary outcome was the total change in lung impedance after 5 min of apnoea compared to a baseline at the beginning of apnoea measured by EIT, normalized to the impedance amplitude during mechanical ventilation at 6–8 mL kg^−1^. This was also analyzed separately in anterior and posterior regions as a secondary outcome. Further secondary outcomes were differences in time to 25%, 50%, and 75% of total impedance change from baseline, and differences in the change of transcutaneous CO_2_ and NIRS from start to end of apnoea between groups.

The primary outcome was analyzed for non‐inferiority for all the intervention groups, using a two‐sided 95% CI (corresponding to a one‐sided alpha level of 0.025) for the mean difference of the low‐flow (0.2 L kg^−1^ min^−1^) group and control (4 L kg^−1^ min^−1^), the high‐flow with normal cannula (2 L kg^−1^ min^−1^) group and control, and for the high‐flow with Switch cannula (2 L kg^−1^ min^−1^) group and control. The non‐inferiority margin was defined a priori as 35% of the global reduction in lung impedance observed in the control group, corresponding to approximately a 10% reduction in functional residual capacity (FRC), which represents the threshold below which clinically relevant atelectasis may be measured. If the upper limit of the two‐sided 95% CI was lower than the non‐inferiority margin, non‐inferiority was assumed.

The sample size of 27 patients per group was based on a simulation study (alpha = 0.05, power = 80%) that used data from our previous study on adults [21], including two patients per group to account for dropouts and missing data.

Normal distributed data are presented as means and standard deviations; otherwise, medians and interquartile ranges are used. Proportions are presented as numbers and percentages. Analytical statistics used Mann–Whitney *U*‐tests and Students *t*‐test, according to distribution, or ANOVA and Kruskal‐Wallis for multiple groups. For traditional group comparisons, a *p* value of < 0.05 was considered statistically significant.

The statistical analyses were performed with R version 4.0.2.

## 3 Results

**FIGURE 1:**
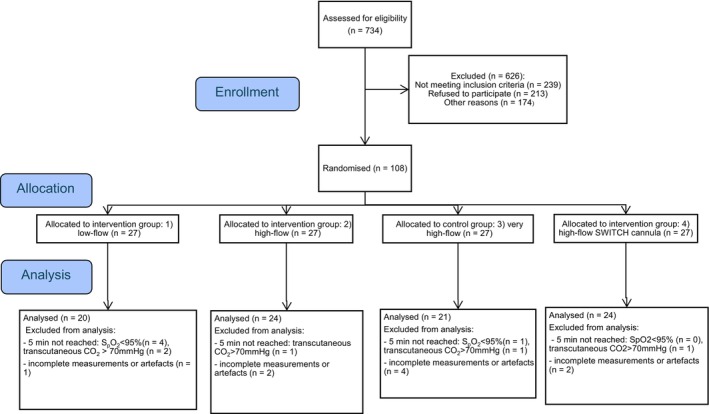
Consolidated standards of reporting trials flow diagram.

We included 108 participants of the study and 89 went into the analysis (Figure 1). Five participants were excluded due to incomplete measurements or major artifacts in the EIT signal, and 10 patients reached a predefined study termination criterion before reaching 5 min of apnoea. Baseline characteristics were comparable between groups as shown in Table 1.

**TABLE 1:** Baseline characteristics of participants according to the flow rate and interface. All participants received humidified and heated 100% oxygen delivered with a high‐flow system.

	Low‐flow 0.2 L kg^−1^ min^−1^	High‐flow 2 L kg^−1^ min^−1^	Very high‐flow 4 L kg^−1^ min^−1^ (control group)	Early onset high‐flow 2 L kg^−1^ min^−1^
	*N* = 20	*N* = 24	*N* = 21	*N* = 24
Age (months)	45 [22;60]	38 [26;50]	32 [27;56]	31 [21;52]
Sex (female)	5 (25%)	8 (33%)	5 (24%)	7 (29%)
Weight (kg)	15 [13;18]	14 [13;16]	15 [12;17]	15 [12;18]
Height (cm)	96 [84;108]	98 [86;107]	97 [88;105]	95 [82;108]
BMI (kg m^−2^)	16.2 [15.1;19.5]	15.6 [14.8;16.6]	15.3 [14.4;17.6]	16.6 [15.7;17.8]
ASA physical status				
I	17 (85%)	20 (83%)	18 (86%)	19 (79%)
II	3 (15%)	4 (17%)	3 (14%)	5 (21%)

*Note:* Data given in median (Q1, Q3) or number (%).

### 3.1 Primary Outcome

**FIGURE 2:**
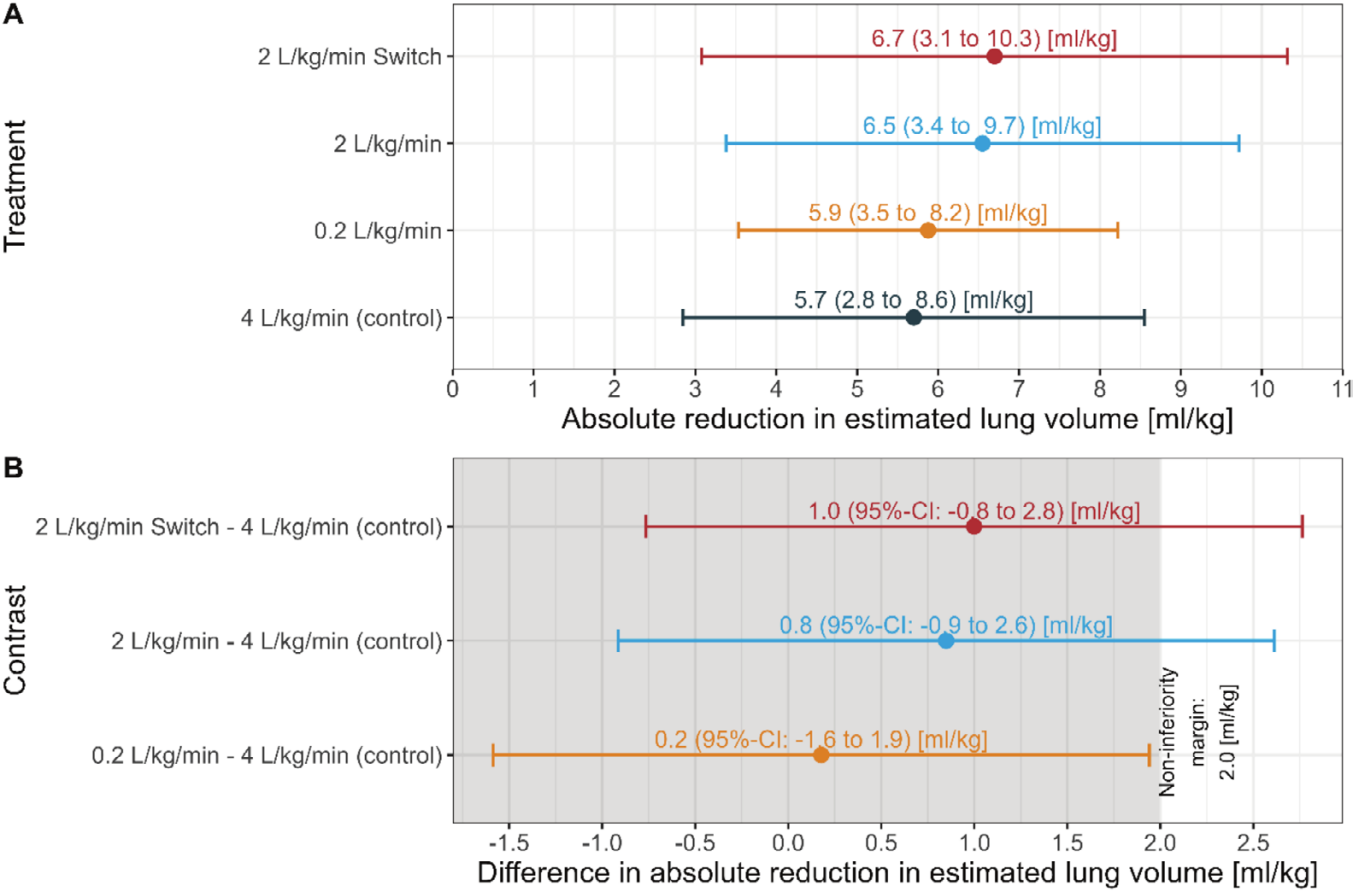
Difference in mean lung volume loss (ml kg^−1^) of intervention groups compared to control (4 L kg^−1^ min^−1^). Non‐inferiority margin (red). Solid dots denote mean values, whereas the errorbars denote ±1 standard deviation (panel A) and 95% confidence intervals (panel B).

Figure 2 shows lung volume reductions across groups; only the low‐flow group met non‐inferiority criteria. The upper limit of the two‐sided 95% CI of the mean difference in reduction of lung volume between the intervention groups high‐flow with late onset of nasal apnoeic oxygenation and high‐flow with early onset of nasal apnoeic oxygenation compared to the control group was higher than the predetermined non‐inferiority margin of 35% of global reduction in lung volume (1.93 mL kg^−1^) of the control group.

### 3.2 Secondary Outcomes

**FIGURE 3:**
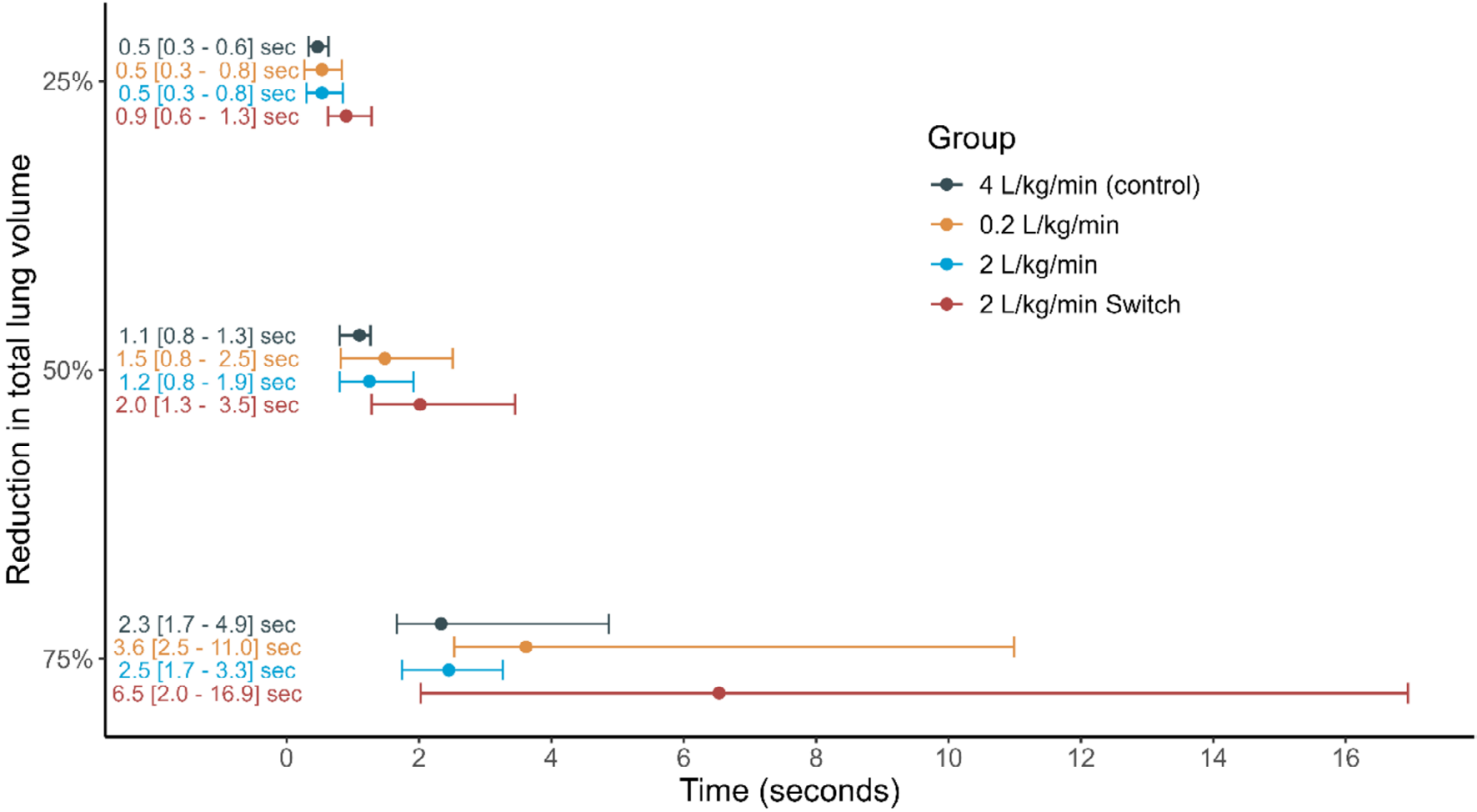
Time to 25%, 50%, and 75% reduction of the total reduction of lung volume per kg bodyweight (mL kg^−1^).

No significant difference in reduction of lung volume could be found when analyzing the two regions of interest: anterior (*p* = 0.7) and posterior (*p* = 0.8). Time to 25% (*p* = 0.7), 50% (*p* = 0.3) and 75% (*p* = 0.12) reduction of the total reduction of lung volume per kg bodyweight (mL kg^−1^) after start of apnoea also did not differ significantly between groups (Figure 3). Time to 75% reduction overall was 2.3 s for the control group, 3.6 s for the low‐flow group, 2.5 s for the high‐flow group, and 6.5 s for the high‐flow Switch cannula group.

## 4 Discussion

This trial evaluated the impact of HFNO flow rate and interface on lung volume loss during apnoea in children. Low‐flow HFNO was non‐inferior to very high‐flow, while moderate‐flow rates (2 L kg^−1^ min^−1^) did not meet non‐inferiority criteria.

Although non‐inferiority could not be shown for all the groups and the different cannulas, we did not find significant differences in loss of lung volume between the investigated flow rates. Thus, it seems that a positive subglottic pressure, enough to reduce lung volume loss during apnoeic oxygenation with an open mouth, cannot be generated regardless of applied flow rates or regardless of the type of the cannula used. These findings are in line with a study performed in adults [20]. A similar study in adult patients conducted by Riedel et al. found no significant difference in lung volume loss using different flow rates of apnoeic oxygenation [21]. Furthermore, adults receiving apnoeic oxygenation with an open mouth show only a minimal airway pressure increase (0.01 cmH_2_O per 10 L min^−1^) [20], which does not prevent atelectasis formation. Although we did not measure the pressure in this study for obvious risks, the results seem to confirm that in pediatric patients, like in adult patients, apnoeic oxygenation with HFNO does not generate positive airway pressures in the trachea. Therefore, also in pediatric patients, the generation of a positive airway pressure cannot be counted as one of the physiological factors contributing to prolonging the apnoea phase. Cardiac oscillations and the concentration of the supraglottic gas flow that enters the trachea via ventilatory mass flow represent the main factors contributing to the maintenance of oxygenation during the apnoea phase [27]. The relatively large variance observed across all groups may reflect inter‐individual differences in respiratory system compliance and lung volume dynamics under anesthesia, rather than differences attributable to the interventions themselves, as the variability was similar across treatment arms.

Most lung volume loss occurred within seconds of apnea onset, consistent with rapid atelectasis formation in children. Although the Switch cannula delayed this loss slightly, the difference was not clinically meaningful given the overall rapid speed of collapse. In contrast, it took adults approximately 50 s for a comparable percentage of reduction [21]. The higher compliance of the chest wall and the greater impact of loss of respiratory muscle tone after induction lead to a faster loss of lung volume in pediatric patients [28, 29]. A further loss of lung volume may occur due to atelectasis formation as the lung volume falls below the closing capacity. Besides other factors such as high oxygen consumption due to a high metabolic rate, this rapid reduction may explain the short time to desaturation after onset of apnoea in children. We can therefore conclude that there is no benefit in rushing the intubation if the child is under apnoeic oxygenation, as the great majority of lung volume loss occurs within seconds.

Limitations of our study are the single‐center study design and the number of excluded patients, which prevented the study from reaching the predefined sample size. Despite recognizing the susceptibility of EIT to artifacts during apnoea, as it was developed to measure tidal ventilation [22], we nonetheless underestimated the drop‐out rate. A direct tracheal pressure measurement would have been ideal, yet not feasible. In our study population, it would have posed an untenable risk. Given the high rate of early termination in the low‐flow group, these results should be interpreted with caution, as the reduced sample size increases uncertainty and limits the strength of evidence for this intervention.

## 5 Conclusions

In summary, low‐flow oxygen was non‐inferior to high‐and very high‐flow in preventing lung volume loss during pediatric apnoea. Our findings suggest that HFNO does not generate clinically relevant airway pressure under these conditions. However, early onset of HFNO may help delay lung volume reduction.

## Author Contributions

Study design, conduct, analysis, manuscript preparation, and supervision: A.F., TRie, TRiv. Patient recruitment, conduct of the study, manuscript preparation: J.A., V.B. Statistical analysis: S.A.‐N., M.H. Study design and finalizing the manuscript: R.G., N.D., G.K.‐M.

## Conflicts of Interest

The authors declare no conflicts of interest.

## Data Availability

The data that support the findings of this study are available on request from the corresponding author. The data are not publicly available due to privacy or ethical restrictions.
